# Identification and Characterization of lncRNAs Related to the Muscle Growth and Development of Japanese Flounder (*Paralichthys olivaceus*)

**DOI:** 10.3389/fgene.2020.01034

**Published:** 2020-09-09

**Authors:** Shuxian Wu, Jingru Zhang, Binghua Liu, Yajuan Huang, Siping Li, Haishen Wen, Meizhao Zhang, Jifang Li, Yun Li, Feng He

**Affiliations:** ^1^The Key Laboratory of Mariculture, Ministry of Education, Ocean University of China, Qingdao, China; ^2^Fisheries College, Ocean University of China, Qingdao, China

**Keywords:** Japanese flounder, skeletal muscle, lncRNA, integrated analysis, transcriptome

## Abstract

Long noncoding RNAs (lncRNAs) play an important role in many life activities, but the expression pattern and function of lncRNAs in Japanese flounder skeletal muscle are unclear. In this study, 751 lncRNAs were selected from skeletal muscle in different development stages of the Japanese flounder [stage A: larval 7 days post hatching (dph); stage B: juvenile about 90 dph; stage C (female) and stage D (male): adult about 24 months] using coding potential analysis methods. In total, 232, 211, 194, 28, 29, and 14 differentially expressed lncRNAs and 9549, 8673, 9181, 1821, 1080, and 557 differentially expressed mRNAs were identified in comparisons of A versus B, A versus C, A versus D, B versus C, B versus D, and C versus D, respectively. We identified the *cis*- and *trans*-regulatory target genes of differentially expressed lncRNAs, and lncRNA–gene interaction networks were constructed using the Cytoscape program. In total, there were 200, 200, 200, 93, 47, and 11 *cis*-regulation relationships, and 29, 19, 24, 38, 8, and 47 *trans*-regulation relationships in the comparisons between A versus B, A versus C, A versus D, B versus C, B versus D, and C versus D, respectively. These results indicate that lncRNA may participate in the development of Japanese flounder skeletal muscle through *cis*- or *trans*-acting mechanisms, thus providing a scientific basis for further study of the biological function of lncRNA in Japanese flounder skeletal muscle. Based on these relationships, functional annotation of the related lncRNAs was performed by gene ontology and Kyoto Encyclopedia of Genes and Genomes (KEGG) enrichment analysis. Differentially expressed genes associated with muscle development were enriched in multiple pairs of comparisons (e.g., differentially expressed genes LOC109640370, LOC109634180, LOC109643555, rusc1, and LOC109626999 were enriched in the actin-binding term, and differentially expressed genes LOC109640370, was, LOC109644970, LOC109643555, and itga9 were enriched in the regulation of the actin cytoskeleton pathway in the KEGG pathway analysis in the comparison of stages C and D). We predicted lncRNA–mRNA, miRNA–mRNA, and lncRNA–miRNA regulatory relationships and constructed interactive networks using Cytoscape software. Co-expression networks show that most lncRNAs interact with one or two predicted miRNAs involved in muscle growth and development. These results provide a basis for further study of the function of lncRNAs on skeletal muscle in different developmental stages of Japanese flounder.

## Introduction

LncRNAs are defined as transcripts that are more than 200 nucleotides in length and are not translated into proteins (Perkel, 2013). This length limitation distinguishes long ncRNAs from small noncoding RNAs, such as microRNAs (miRNAs), small interfering RNAs (siRNAs), Piwi interacting RNAs (piRNAs), small nucleolar RNAs (snoRNAs), and other short RNAs (Lina et al., 2013). Note that only one fifth of transcriptions are associated with protein-coding genes in the human genome (Philipp et al., 2007). Large-scale cDNA library sequencing and transcriptome sequencing indicate that tens of thousands of intergenic sites are transcribed to noncoding RNAs in mammals. Approximately 78% of lncRNAs are tissue specific, and only ∼19% of mRNAs are tissue specific (Cabili et al., 2011). At present, more and more lncRNAs have been found in mammals, such as humans (Pieter-Jan et al., 2013; Iyer et al., 2015), mice (Phillip et al., 2013; Lv et al., 2015), and sheep (Bakhtiarizadeh et al., 2016) as well as in plants, such as rice (Zhang et al., 2014). It has been reported that lncRNA plays an important role in the regulation of gene transcription (Goodrich and Kugel, 2006), post-transcriptional regulation (Ming-De et al., 2005), epigenetic regulation (Mercer and Mattick, 2013; Morlando et al., 2014), and aging and disease (Lukiw et al., 1992). Although there is growing evidence that most of them may be functional (Mercer et al., 2009), only a relatively small proportion has been shown to be biologically relevant.

Skeletal muscle is a striated muscle tissue composed of muscle cells with contractile capacity. It is well known that the fetal stage is the main stage of skeletal muscle development, and there is no net increase in the number of muscle fibers after birth (Nishina et al., 2003). Muscle development is a complex process that requires interactions between multiple factors (Buckingham, 2006). At present, studies on skeletal muscle growth and development generally focus on the expression and function of related coding genes (Thomas and Mathias, 2011; Eng et al., 2013). The skeletal muscle fiber phenotype is regulated by various independent signaling pathways, including the mitogen-activated protein kinase (MAPK) pathway (Keren et al., 2006), calcineurin (Naya et al., 2000), calcium/calmodulin-dependent protein kinase IV (Takayuki et al., 2004), and the peroxisome proliferator γ coactivator 1 (PGC-1) (Handschin et al., 2003). Studies show that some miRNAs are also involved in the development of skeletal muscle (Tang et al., 2015; Jebessa et al., 2018). Several recent studies show that lncRNAs also play a crucial role in skeletal muscle development (Zhan et al., 2016; Liang et al., 2017; Zhou et al., 2018). In addition, lncRNAs can interact as a competitive endogenous RNA (ceRNA) with miRNAs involved in the regulation of target gene expression, thereby regulating muscle development (Cesana et al., 2011).

Japanese flounder is a valuable marine fish and an important economic fish for marine aquaculture in Asia. Therefore, it is important to reveal the molecular mechanisms of Japanese flounder skeletal muscle formation and development. Studies show that some coding genes play important roles during the development of Japanese flounder skeletal muscle (Huang et al., 2018; Wu et al., 2018). In addition, some studies focus on the effects of noncoding RNA on skeletal muscle development of Japanese flounder. Some micRNAs (mir-1, mir-133, mir-206) play an important role in muscle development during Japanese flounder metamorphosis (Fu et al., 2011, 2012). However, information on lncRNAs related to skeletal muscle development in the Japanese flounder is still limited.

In this study, we used the Illumina HiSeq 2500 platform to identify lncRNAs and mRNAs involved in skeletal muscle development in Japanese flounder. Our study provides useful information for further study of the function of lncRNA during skeletal muscle development in a fish species, and these results will help study skeletal muscle development from the perspective of noncoding RNAs.

## Materials and Methods

### Ethics Statement

The study was approved by the respective Animal Research and Ethics Committees of Ocean University of China. The field studies did not involve endangered or protected species. The fish were all euthanized by tricaine methanesulfonate (MS-222) prior to experimentation.

### Experimental Animal Collection

Japanese flounder were collected from the Donggang District Institute of marine treasures in Rizhao, Shandong province. The fish were transported to the Ocean University of China and temporarily reared in a 500-L white bucket for 24 h. The Japanese flounder were collected at various stages: larval 7 days post hatching (dph) (stage A), juvenile ∼90 dph (stage B), female adult ∼24 months (stage C), and male adult ∼24 months. In our experiment and data analysis, 3 fish were used in all stages except for stage A (here fish were very small in size, so ∼50 individuals were combined and considered to be one sample). All fish were euthanized with MS-222, and tissue samples were collected. In stage A, we used a microscope to cut off redundant tissue and only retain muscle tissue. Samples were immediately frozen in liquid nitrogen and then stored at −80°C until further processing.

### Illumina Deep Sequencing and Sequence Analysis

Total RNA for RNA sequencing (RNA-seq) was extracted using TRIzol Reagent (Invitrogen, Carlsbad, CA, United States) according to the manufacturer’s protocol. The concentration of RNA was quantified by the nucleic acid analyzer Biodropsis BD-1000 (OSTC, China) and the integrity by agarose gel electrophoresis examination. Ribosomal RNA (rRNA) was removed from the total RNA using the Epicenter Ribo-Zero^TM^ rRNA Removal Kit (Epicenter, Madison, WI, United States) following the manufacturer’s instructions. The constructed cDNA library was quality tested on an Agilent Bioanalyzer 2100 system, and then high-throughput sequencing was performed on the Illumina HiSeqTM 2500 platform. The paired-end sequencing raw reads were cleared by removing reads containing adapters, including ploy-N reads and low-quality reads to obtain clean reads. At the same time, the Phred score (Q20), Q30, and GC contents of the clean data were calculated. All the downstream analyses were based on the high-quality clean data. The clean reads were mapped to the Japanese flounder reference genome^1^ using the Tophat2 software.

**FIGURE 1:**
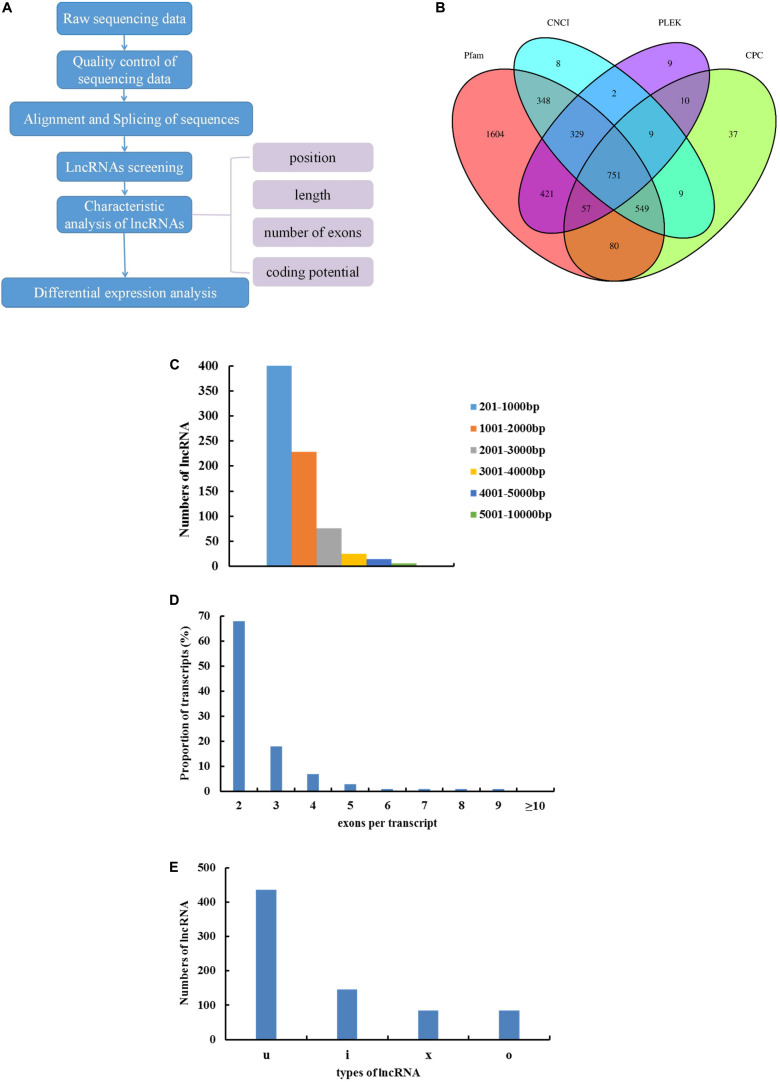
The features of Japanese flounder lncRNAs. **(A)** Identification and verification of lncRNA in skeletal muscle of Japanese Flounder. **(B)** Venn diagram of Candidate lncRNA coding ability prediction result. **(C)** Exon length distribution of Japanese flounder lncRNAs. **(D)** Exon numbers per transcript of Japanese flounder lncRNAs. **(E)** The lncRNAs types of Japanese flounder skeletal muscle, “I” (intergenic lncRNA), “u” (intronic lncRNA), “x” (anti-sense lncRNA), “o” (sense-overlapping lncRNA).

Reconstructing transcripts for clean readings was based on probabilistic models using Cufflinks 2.0.2 software. Based on the characteristics of lncRNA, we used a rigorous three-step screening method to obtain candidate lncRNAs (Figure 1A). First, Cuffcompare software was used to screen out transcripts that were perfectly matched or similar to other ncRNAs, mRNAs, etc., while clarifying the location type of the remaining transcripts. We then retained transcripts annotated as “i” (intergenic lncRNA), “u” (intronic lncRNA), “x” (anti-sense lncRNA), and “o” (sense-overlapping lncRNA) by screening for candidate lncRNA transcripts. Second, single-exon transcripts and transcripts < 200 bp long were removed. Finally, we used four analytical tools, including CPC (encoding potential calculator) (Lei et al., 2007), CNCI (coding-non-coding-index) (Liang et al., 2013), Pfam Scan (Finn et al., 2014), and PLEK (Li et al., 2014) to predict the coding potential of the transcripts. CPC score ≤ 0, CNCI score ≤ 0, Pfam: *E*-value ≤ 0.001, and coding_potential_score ≤ 0 were conditions for screening lncRNA. The transcript expression levels were calculated using the fragments per kb per million (FPKM) reads method, which is the number of fragments per kilobase length from a gene per million fragments. The transcript differential expression was calculated according to the negative binomial distribution test in the DESeq (Anders and Huber, 2012) software^2^. Transcripts with *p* < 0.05 and | (fold change) | ≥ 2 were designated as differentially expressed. The sequencing data obtained from RNA-seq were released to the National Center for Biotechnology Information (NCBI) Sequence Read Archive (SRA) database under the accession numbers SRR12102079, SRR12102078, SRR12102077, SRR12102076, SRR12102075, SRR12102074, SRR12102073, SRR12102072, SRR12102071, SRR12102070, SRR12102069, and SRR12102068.

### *Cis*- and *Trans*-Analyses and Enrichment Analysis

We searched for all of the coding genes 100 kb upstream and downstream of differentially expressed lncRNA that had significant co-expression (Pearson correlation calculation) with the lncRNA. These genes that are genomically adjacent and coexpressed in the expression pattern are likely to be the *cis*-target genes of the lncRNA. Based on the results of differential co-expression, lncRNA and mRNA not on the same chromosome were selected as candidate targets. The RNA interaction software RIsearch-2.0 was used to predict the binding of candidate lncRNA and mRNA at the nucleic acid level. The number of bases in which two nucleic acid molecules directly interact with each other is not less than 10 and the free energy of base binding is not more than −50 were used as screening conditions, and they determined the potential that the lncRNA was *trans*-acting. Differentially expressed lncRNAs and their corresponding differentially expressed *cis*- and *trans*-target genes were used to construct lncRNA–gene interaction networks using the Cytoscape program. Predicting the main functions of lncRNA was done by functional enrichment analysis of lncRNA target mRNA genes. We performed gene ontology (GO) enrichment analysis (Young et al., 2010). The number of differential transcripts included in each GO entry was counted, and the significance of differential transcript enrichment in each GO entry was calculated using the hypergeometric distribution test method. The Kyoto Encyclopedia of Genes and Genomes (KEGG) (Minoru et al., 2008) is the main public database of Pathway and is used to perform Pathway analysis on differential transcripts (combined with KEGG annotation results). The significance of differential transcript enrichment in each Pathway entry is calculated by a hypergeometric distribution test.

### Quantitative Real-Time-PCR (qRT-PCR) Analysis

Four differentially expressed lncRNAs target genes that affect muscle development were selected for qRT-PCR verification, including calcium voltage-gated channel subunit alpha1 D (cacna1d) (Krasnyi and Ozernyuk, 2011), actin-related protein 3B-like (Tseng et al., 2002), kin of IRRE like 2 (kirrel2) (Durcan et al., 2014), myosin-7B-like (Girgenrath et al., 2005), and their corresponding lncRNA regulatory factors (TCONS_00034769, TCONS_00041871, TCONS_00089031, and TCONS_00038291). The qRT-PCR primers for these lncRNAs and genes are shown in Table 1. Quantitative real-time PCR was conducted with the Roche LightCycler480 (Germany) and SYBR Premix Ex Taq^TM^ (TliRNaseH Plus) Kit (Takara, Japan) to determinate the relative expressions of each gene. Primers are listed in Table 1, and the Japanese flounder 18 s (GenBank accession no. EF126037.1) was used as the endogenous reference gene. The amplified system was formed from 10μL SYBR^®^Premix Ex Taq (TliRNaseH Plus), 0.4μL ROX reference dye, 0.4μL PCR forward primer, 0.4μL PCR reverse primer, 2μL cDNA template, and 20 μL of RNase-free water. The reaction was completed according to the following procedure: 95°C for 30 s, 40 cycles of 95°C for 5 s, and Tm for 30 s. All samples were run in triplicate. Then, we calculated the relative expression by the method of comparative threshold (2−ΔΔCt) (Livak and Schmittgen, 2001).

**TABLE 1:** Primers used for real time PCR.

**LncRNA and gene**	**Primer sequence (5′-3′)**	**Product length (bp)**	**Annealing temperature (°C)**
TCONS_ 00041871	F: CTCCTGAACCCTTTTCTCCT R:GCTCAGTCTGACTTTAGTGCC	153	60
TCONS_ 00034769	F: ACTGCTCTGGCCTGAGGATG R: CGGCTCTATTGTGGGGAACC	198	65
TCONS_ 00089031	F: CTCACTGTGGGTTTTCAAGC R: TTTGAGCCAGAACAGAGGGT	173	65
TCONS_ 00038291	F:GACGCAGAGGAAAGAAGCAC R: GGAGCAACTTCCTCAGACCT	176	65
Actin-related protein 3B-like	F:TGAGTGGAGGACGGATAAAG R: TCGGACCAATCTCATCGTAG	159	60
Cacna1d	F: ACGCTACTCTGTTTGCTCTG R:AACTTCCCCACTGTTACCTC	178	60
Kirrel2	F: CGTGGTGCTCAGTAATGGTA R: CGTCTGCTGTGATGATAGGT	155	60
Myosin-7B-like	F:AGATTGAGGGGATAGAGTGG R: CCCAGATGGTTGTCATAGAG	169	60
18S	F:ATTGACGGAAGGGCACCAC R:ATGCACCACCACCCACAGA	134	65

### LncRNA–miRNA–mRNA Network Construction

Cytoscape is an open source software for biological network integration, visualization, and analysis, loading molecular and genetic interaction data sets in many standard formats in the fields of molecular and systems biology, genomics, and proteomics (Saito et al., 2012). In this study, lncrRNA–mRNA, miRNA–mRNA, and lncRNA–miRNA relationship pairs with regulatory relationships were predicted, and Cytoscape software was used to construct the interaction network diagram among them. The nodes in the network diagram are the mRNAs related to muscle development in the GO enrichment and KEGG pathways; the lncRNAs that have a regulatory relationship with mRNAs; the miRNAs that have a regulatory relationship with mRNAs. The sequencing data obtained from miRNA-seq were released to the NCBI SRA database under the accession numbers SRR11968806, SRR11968805, SRR11968804, SRR11968803, SRR11968802, SRR11968801, SRR11968800, SRR11968799, SRR11968798, SRR11968797, SRR11968796, and SRR11968795.

### Statistical Analysis

The data were presented as means ± SEM. The statistical differences were analyzed by one-way ANOVA and Duncan’s multiple range tests in SPSS 19.0 software. *P* < 0.05 was considered to be statistically significant.

## Results

### Overview of RNA-Sequencing

High-throughput RNA-seq was performed on the Illumina Hiseq 2500 platform. Each library produced more than 90 million raw reads. After filtering low-quality reads, clean reads still accounted for more than 93% of the raw reads. More than 67.32% of the clean reads perfectly mapped to the reference genome of the Japanese flounder. The uniquely mapped reads ranged from 62.16 to 75.55% of the clean reads (Table 2).

**TABLE 2:** Summary of draft reads of 12 libraries by RNA-sequencing.

**Sample**	**Raw reads**	**Clean reads**	**Total mapped**	**Multiple mapped**	**Uniquely mapped**
A_1_1	95,070,396	90,140,748 (94.81%)	68,921,334 (76.46%)	2,510,198 (2.78%)	66,411,136 (73.67%)
A_1_2	95,386,604	90,562,700 (94.94%)	71,317,689 (78.75%)	2,896,374 (3.20%)	68,421,315 (75.55%)
A_1_3	98,029,082	92,699,826 (94.56%)	72,020,838 (77.69%)	2,679,887 (2.89%)	69,340,951 (74.80%)
B_2_1	96,622,226	91,173,512 (94.36%)	67,805,686 (74.37%)	6,443,523 (7.07%)	61,362,163 (67.30%)
B_2_2	96,239,174	91,922,114 (95.51%)	66,710,930 (72.57%)	5,541,344 (6.03%)	61,169,586 (66.55%)
B_2_3	96,343,382	90,217,708 (93.64%)	60,737,386 (67.32%)	4,662,151 (5.17%)	56,075,235 (62.16%)
C_3_1	95,721,552	91,153,382 (95.23%)	71,011,936 (77.90%)	10,055,963 (11.03%)	60,955,973 (66.87%)
C_3_2	95,830,852	91,285,636 (95.26%)	72,297,477 (79.20%)	1,278,4703 (14.01%)	59,512,774 (65.19%)
C_3_3	97,999,946	93,132,198 (95.03%)	73,007,033 (78.39%)	12,142,118 (13.04%)	60,864,915 (65.35%)
D_4_1	98,391,502	92,247,994 (93.76%)	65,722,331 (71.25%)	8,110,885 (8.79%)	57,611,446 (62.45%)
D_4_2	98,380,634	93,523,450 (95.06%)	73,108,525 (78.17%)	10,076,310 (10.77%)	63,032,215 (67.40%)
D_4_3	96,840,342	92,390,402 (95.40%)	73,089,193 (79.11%)	9,858,949 (10.67%)	63,230,244 (68.44%)

### Identification of lncRNAs in Japanese Flounder Skeletal Muscle

According to the characteristics of lncRNAs, RNA-seq produced 751 lncRNA transcripts after strict screening and filtering of RNAs that did not meet the requirements (Figure 1B). The length of lncRNAs ranged from 201 to 9381 bp; the length of lncRNAs between 201 and 1000 bp was 53.3%, 1000–2000 bp was 30.5%, and the average length of lncRNAs was 1243 bp (Figure 1C). The lncRNAs with 2 exons were 68% and with 3 exons were 18% (Figure 1D). The number of predicted lncRNAs types was 436 for intergenic lncRNA (u), 146 for intronic lncRNA (i), 85 for anti-sense lncRNA (x), and 84 for sense-overlapping lncRNA (o) (Figure 1E).

### Differential Expression Analysis of lncRNAs and mRNAs

**FIGURE 2:**
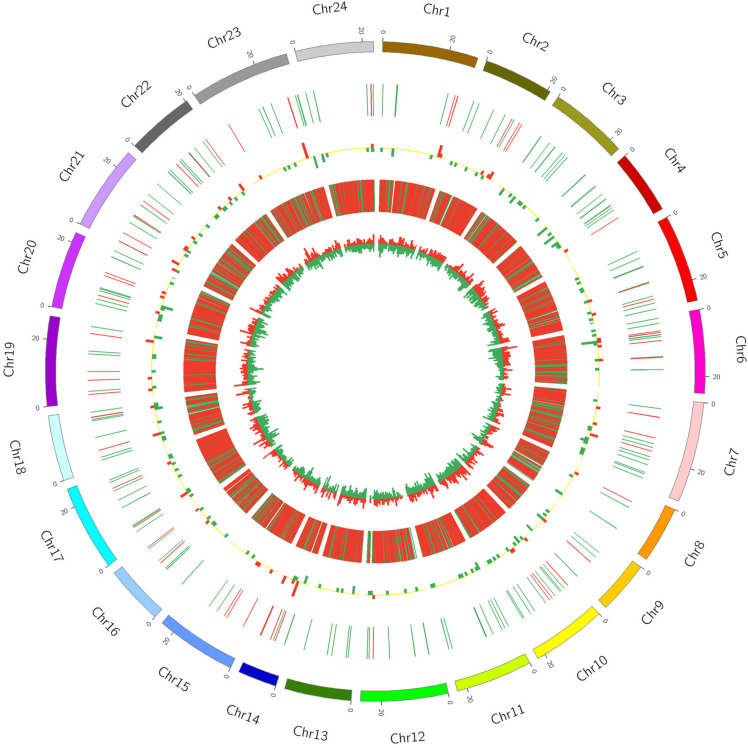
Circos diagram of differential expression of lncRNA and mRNA in A vs. B. In the figure, the outermost circle is the autosomal distribution of the Japanese flounder; the second circle is the lncRNA of differential expression on the chromosome, the red line indicates up-regulation, the green line indicates down-regulation; the third circle is the histogram of differentially expressed lncRNAs at different positions. Red indicates up-regulation, green indicates down-regulation, and the higher the column, indicates the more differentially expressed gene numbers. The fourth circle is the distribution of differentially expressed mRNAs on the chromosome, and the color distribution is the same as lncRNA; the innermost circle is the column with differentially expressed mRNAs at different positions, color distribution is the same as lncRNA.

In order to display the information on differentially expressed lncRNAs and mRNAs more intuitively, the differential expression of lncRNAs and mRNAs in the same differential comparison group was shown by using Circos software (Figure 2 and Supplementary Figure S1). In the A versus B comparison, 232 differentially expressed lncRNAs were detected, 67 of which were upregulated and 165 were downregulated. A total of 9549 differentially expressed mRNAs were detected, 3041 of which were upregulated and 6508 were downregulated (Figure 2 and Supplementary Table S1). In the A versus C comparison, 211 differentially expressed lncRNAs were detected, 60 of which were upregulated and 151 were downregulated. A total of 8673 differentially expressed mRNAs were detected, 2665 of which were upregulated and 6008 were downregulated (Supplementary Figure S1 and Supplementary Table S2). In the A versus D comparison, 194 differentially expressed lncRNAs were detected, 63 of which were upregulated and 131 were downregulated, and 9181 differentially expressed mRNAs were detected, 2854 of which were upregulated and 6327 were downregulated (Supplementary Figure S1 and Supplementary Table S3). In the B versus C comparison, 28 differentially expressed lncRNAs were detected, 13 of which were upregulated and 15 were downregulated, and 1821 differentially expressed mRNAs were detected, 978 of which were upregulated and 843 were downregulated (Supplementary Figure S1 and Supplementary Table S4). In the B versus D comparison, 29 differentially expressed lncRNAs were detected, 12 of which were upregulated and 17 were downregulated, and 1080 differentially expressed mRNAs were detected, 532 of which were upregulated and 548 were downregulated (Supplementary Figure S1 and Supplementary Table S5). In the C versus D comparison, 14 differentially expressed lncRNAs were detected, 7 of which were upregulated and 7 were downregulated, and 557 differentially expressed mRNAs were detected, 245 of which were upregulated and 312 were downregulated (Supplementary Figure S1 and Supplementary Table S6).

### lncRNA–Gene Interaction Network Construction

**FIGURE 3:**
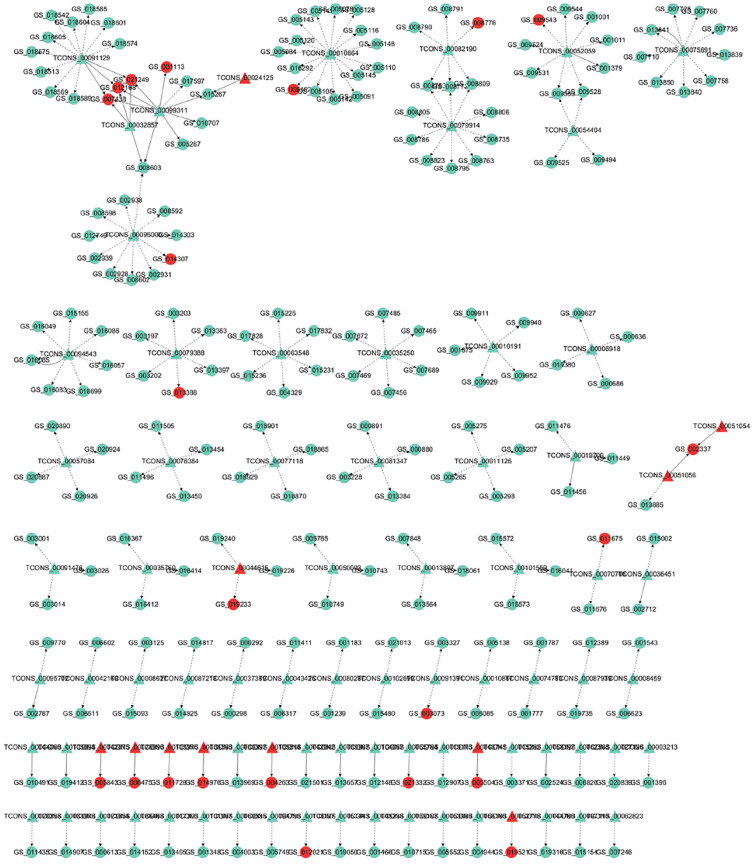
LncRNA – gene interaction network diagram in A vs. B. Red indicates up-regulation, green indicates down-regulation, triangles represent lncRNA, and circles indicate mRNA. The dashed line indicates the interaction between the differentially expressed lncRNA and its corresponding cis target gene, while the solid line indicates the interaction between the differentially expressed lncRNA and its corresponding trans target gene.

To address how lncRNA interacts with its target gene (mRNA) to regulate Japanese flounder muscle development and identify key molecular players in the process, we predicted *cis*- and *trans*-targets of differentially expressed lncRNAs and constructed the possible regulatory networks for these interactions. Previous studies have demonstrated that lncRNAs regulate the expression of adjacent protein-coding genes via a *cis*-acting mechanism (Han et al., 2012; Qian et al., 2012). In the present study, we screened for all the coding genes in the 100k that were upstream and downstream of the differentially expressed lncRNAs and significantly coexpressed with the lncRNAs (Pearson correlation calculation, Supplementary Tables S14–S19). These genes that are genomically adjacent and coexpressed in expression patterns are predicted to be *cis*-target genes of lncRNAs. In addition, lncRNAs regulate the expression of genes located on other chromosomes through a *trans*-acting mechanism (Han et al., 2012). Based on the results of differential co-expression, the lncRNAs and mRNAs that are not on the same chromosome were selected as candidate targets. The RNA interaction software RIsearch-2.0 was used to predict the binding of candidates of lncRNA and mRNA at the nucleic acid level. The lncRNAs and mRNAs that may have direct regulation were screened, and these genes were predicted to be *trans*-target genes. For the comparison of A and B, the lncRNA-gene interaction network contained 304 network nodes, 83 lncRNAs, 221 protein-coding genes, 200 pairs of *cis*-regulation relations, and 29 pairs of *trans*-regulation relations (Figure 3 and Supplementary Table S7). For the comparison of A and C, the lncRNA–gene interaction network contained 285 network nodes, 70 lncRNAs, 215 protein encode genes, 200 pairs of *cis*-regulation relations, and 19 pairs of *trans*-regulation relations (Supplementary Figure S2 and Supplementary Table S7). For the comparison of A and D, the lncRNA–gene interaction network consists of 278 network nodes, 69 lncRNAs, 209 protein-coding genes, 200 pairs of *cis*-regulation relations, and 24 pairs of *trans*-regulation relations (Supplementary Figure S2 and Supplementary Table S7). For the comparison of B and C, the lncRNA–gene interaction network contained 157 network nodes, 26 lncRNAs, 131 protein-coding genes, 93 pairs of *cis*-regulation relations, and 38 pairs of *trans*-regulation relations (Supplementary Figure S2 and Supplementary Table S7). For the comparison of B and D, the lncRNA–gene interaction network contained 108 network nodes, 22 lncRNAs, 86 protein encode genes, 47 pairs of *cis*-regulation relations, and 8 pairs of *trans*-regulation relations (Supplementary Figure S2 and Supplementary Table S7). For the comparison of C and D, the lncRNA–gene interaction network contained 97 network nodes, 12 lncRNAs, 85 protein-coding genes, 11 pairs of *cis*-regulation relations, and 47 pairs of *trans*-regulation relations (Supplementary Figure S2 and Supplementary Table S7). We then analyzed the expression correlation between the network lncRNA and its corresponding target gene. In the network constructed from the differentially expressed lncRNAs and target genes identified from the A versus B comparison, 212 lncRNA–gene linkages were positively correlated, and the other 18 linkages were negatively correlated (Figure 3 and Supplementary Table S7). For the A versus C comparison, 210 lncRNA–gene connections were positively correlated, and 9 connections were negatively correlated (Supplementary Figure S2 and Supplementary Table S7). For the comparison of A and D, 213 lncRNA–gene connections were positively correlated, and 11 connections were negatively correlated (Supplementary Figure S2 and Supplementary Table S7). For the B versus C comparison, 73 lncRNA–gene connections were positively correlated, and 58 connections were negatively correlated (Supplementary Figure S2 and Supplementary Table S7). For the B versus D comparison, 46 lncRNA–gene connections were positively correlated, and 9 connections were negatively correlated (Supplementary Figure S2 and Supplementary Table S7). For the C versus D comparison, 24 lncRNA-gene connections were positively correlated, and 34 connections were negatively correlated (Supplementary Figure S2 and Supplementary Table S7). The directional analysis shows that the positive correlation number between lncRNA–gene pairs was higher than the negative correlation number except for comparison networks of C and D.

### GO and KEGG Pathway Analysis

**FIGURE 4:**
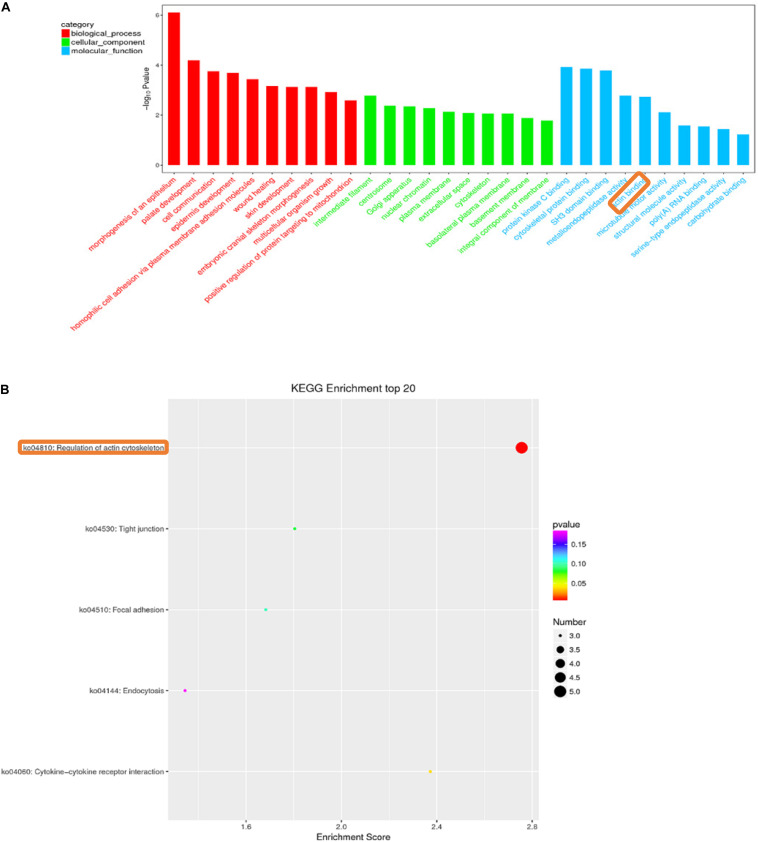
Histogram of gene ontology (GO) classification **(A)**. GO analysis of C vs. D differentially expressed lncRNAs target genes. The horizontal axis indicates the GO entry name and the vertical axis indicates −log10Pvalue. Red bars: biological process; Green bars: cellular component; Blue bars: molecular function. KEGG pathway enrichment of C vs. D differentially expressed lncRNAs target genes **(B)**. The horizontal axis is the enrichment score, the vertical axis indicates the name of the pathway.

We enriched the biological processes and pathways in all comparisons. In the A versus B comparison, 3805 terms were enriched, and 3720 terms were enriched in the A versus C comparison, 3627 terms in A versus D, 1779 terms in B versus C, 1780 terms in B versus D, and 741 terms were enriched in the C versus D comparison (*p* < 0.05). We selected the top 30 terms in the GO enrichment analysis for each comparison (screening GO entries with corresponding transcript numbers greater than 2, sorting from large to small according to the corresponding −log10P-value for each entry and then selecting 10 terms in each of the three categories) analysis (Supplementary Tables S8–S13). Many GO terms that were enriched in more than one comparison were related to myosin filament, epidermal cell differentiation, and calcium- and calmodulin-responsive adenylate cyclase activity. In all of these GO terms, there were enriched muscle-related terms, such as muscle myosin complex, myosin filament, and actin binding. In all of these GO terms, muscle-related terms, such as muscle myosin complex, myosin filament, and actin binding, were enriched. Figure 4A is a GO enrichment map of the C versus D comparison in which actin binding is significantly enriched in the top 30. For A versus B, A versus C, A versus D, B versus C, B versus D, and C versus D comparisons, 79, 85, 72, 31, 31, and 31 enrichment pathways were detected, respectively (*p* < 0.05). We selected the top 20 terms in the KEGG pathway analysis for each comparison (screening the pathway entries with transcript numbers greater than 2 and sorting from large to small according to the corresponding −log10P-value for each entry) (Supplementary Tables S8–S13). The regulation of the actin cytoskeleton pathway was significantly enriched in the C versus D comparison (Figure 4B). These results suggest that some lncRNAs may be involved in the growth and development of skeletal muscle.

### Verification of Gene Expression Profiles Using qRT-PCR

**FIGURE 5:**
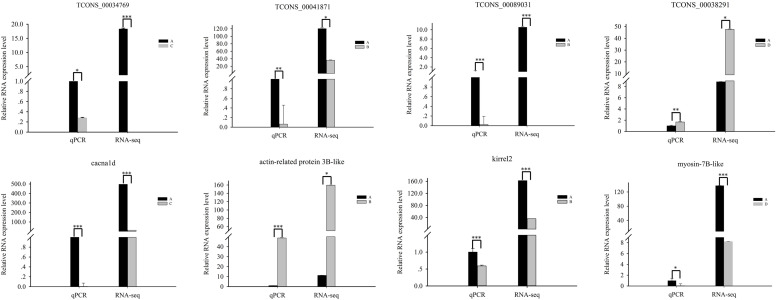
Quantitative real-time PCR validation of differentially expressed lncRNAs and their corresponding target genes. Values are shown as mean with SEM. Significant differences between stages are indicated by *. *, **, and *** indicate *p* < 0.01, *p* < 0.001, and *p* < 0.0001, respectively.

To confirm the accuracy and reproducibility of differentially expressed lncRNAs and differentially expressed gene expression levels obtained from RNA-seq, we selected four differentially expressed lncRNA target genes that affect muscle development and performed qRT-PCR verification. The expression of cacna1d was downregulated in stage C compared with that in stage A. The expression of TCONS_00034769 was downregulated in stage C compared with that in stage A. The expression of actin-related protein 3B-like was upregulated in stage B compared with that in stage A. The expression of TCONS_00041871 was downregulated in stage B compared with that in stage A. The expression of kirrel2 was downregulated in stage B compared with that in stage A. The expression of TCONS_00089031 was downregulated in stage B compared with that in stage A. The expression of myosin-7B-like was downregulated in stage D compared with that in stage A. The expression of TCONS_00038291 was upregulated in stage D compared with that in stage A. All four lncRNAs and their target genes showed similar expression patterns compared to RNA-seq data, indicating the reliability of our RNA-seq data (Figure 5).

### Bioinformatics Analysis of lncRNA–miRNA–mRNA Networks

**FIGURE 6:**
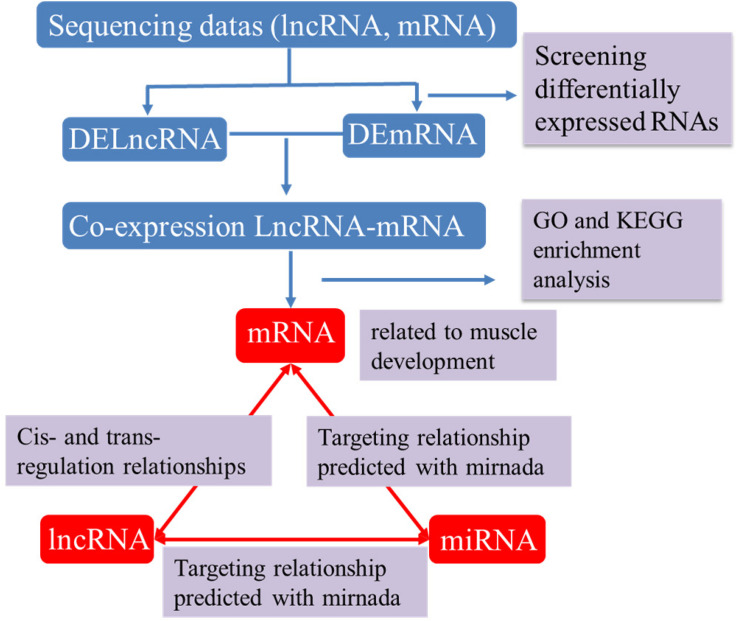
The process of building lncRNA – miRNA-mRNA interactive network. DElncRNA: differentially expressed lncRNA; DEmRNA: differentially expressed mRNA.

**FIGURE 7:**
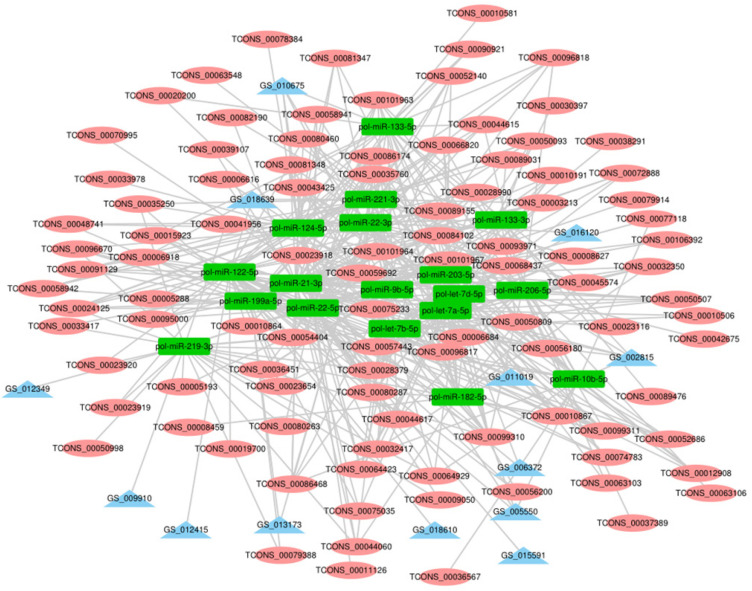
The lncRNA – miRNA-mRNA interaction network in A vs. B. In the network, blue triangles represent mRNAs, red ovals represent lncRNAs, and green rectangles represent miRNAs.

Recent studies show that lncRNAs can act as a competitive endogenous RNA affecting post-transcriptional regulation by interfering with the miRNA pathway. To further elucidate the role of lncRNAs in the growth and development of skeletal muscle in Japanese flounder, we used TargetScan and miRanda to predict miRNAs that have a regulatory relationship with the given muscle development–associated mRNAs and the differentially expressed lncRNAs and established the basic lncRNA linkage (Figure 6). The lncRNA–miRNA–mRNA interaction network (Figure 7, Supplementary Figure S2,S3 and Supplementary Table S7) was constructed using Cytoscape software. In the A versus B comparison, there were 18 miRNAs, 13 mRNAs, and 101 lncRNAs, which had at least one predicted target miRNA. There were 18 miRNAs, 13 mRNAs, and 110 lncRNAs in the A versus C comparison. There were 18 miRNAs, 13 mRNAs, and 95 lncRNAs in the A versus D comparison. There were 19 miRNAs, 13 mRNAs, and 11 lncRNAs in the B versus C comparison. There were 19 miRNAs, 13 mRNAs, and 12 lncRNAs in the B versus D comparison. There were 19 miRNAs, 13 mRNAs, and 4 lncRNAs in the C versus D comparison. Some of these lncRNAs (including TCONS_00003213, TCONS_00006684, and TCONS_00023918) were found to interact with at least three target miRNAs.

## Discussion

More and more lncRNAs have been discovered in different tissues and cells by high-throughput sequencing technology, some of which have been proven to play important roles in the growth or disease development of some mammals (Geng et al., 2013; White et al., 2014; Gao et al., 2017) and other model organisms (Pauli et al., 2012; Grote et al., 2013). In this study, only 67.32% of the B_2_3 sample in the RNA-seq results was mapped to the Japanese flounder genome, and the mapping rate was a bit low. The main reason for the low reference genome-mapping rate is that the genome assembly of the reference species is not ideal. NCBI has two reference genomes of Japanese flounder, and the assembly quality is not high. ContigN50 is 30K and 36K, respectively. In general, the longer the contigN50, the better the assembly result. Previous reports have shown that the mapping rates were only 74 and 85% (Zhang et al., 2016; Sun et al., 2020), which are relatively low. There are still few analyses of Japanese flounder with the reference genome, most of which are Denovo strategies, which also reflects that the Japanese flounder genome is not very good.

The identification and characterization of lncRNAs in Japanese flounder, especially in skeletal muscle development, are very limited compared to those of lncRNAs in mammals. In this study, we identified 751 lncRNAs in four stages (A, B, C, D) of Japanese flounder skeletal muscle development through high-throughput sequencing. We also identified differentially expressed mRNAs and lncRNAs among the A versus B, A versus C, A versus D, B versus C, B versus D, and C versus D comparisons, respectively, 9549, 8673, 9181, 1821, 1080, and 557 differentially expressed mRNAs and 232, 211, 194, 28, 29, and 14 differentially expressed lncRNAs. These may have specific biological functions in skeletal muscle development of Japanese flounder. In recent years, the biological effects of some lncRNAs in muscle have been reported. For example, it is reported that lncRNA AK 017368 can promote the proliferation and inhibit the differentiation of skeletal muscle myoblasts (Liang et al., 2017). Linc-MD1 is the first identified lncRNA specifically involved in muscle differentiation (Cesana et al., 2011). In addition, the newly identified lncRNA MAR promotes muscle differentiation and regeneration and may also be a novel therapeutic target for the treatment of aging or muscle atrophy (Zhang et al., 2018). Therefore, differentially expressed lncRNAs identified here may also affect the development of skeletal muscle in Japanese flounder.

It is well known that lncRNAs can function by targeting protein-encoding genes. In this study, we hypothesized their potential biological function by predicting the *cis*- and *trans*-regulated target genes of lncRNAs. Recent studies have also shown that lncRNAs are involved in *cis*-regulatory activity in muscle development. Studies have shown that lncRNA-Six1, located 432 bp upstream of the protein-encoding gene Six homeobox 1 (Six1), promotes cell proliferation and participates in muscle growth through *cis*-acting regulation of genes (Cai et al., 2017). Previous studies show that the lncRNA Dum located upstream of the developmental pluripotency-associated 2 (Dppa2) gene is involved in myogenic differentiation and muscle regeneration (Wang et al., 2015). Here, we screened all the coding genes 100k upstream and downstream of the differentially expressed lncRNAs as *cis*-targets. In the comparisons of A versus B, A versus C, A versus D, B versus C, B versus D, and C versus D, 200, 200, 200, 93, 47, and 11 pairs of lncRNAs and genes were found to have *cis*-regulation relations, respectively. The identified *cis*-regulatory genes have important significance for predicting the function of lncRNAs. Thus, the regulatory role of lncRNAs on muscle development needs to be investigated further. Many studies have shown that lncRNA participates in muscle development through *trans*-acting. The synergistic activity of the two DNA enhancer elements CE and DRR located in the ∼24 kb upstream region of MYOD1 regulates the level of MyoD expression in the myogenic lineage, whereas DDRRNA promotes the expression of myogenin by *trans*-acting (Mousavi et al., 2013). MUNC, also known as DRR (eRNA), is located 5 kb upstream of the transcription start site of MyoD and promotes the function of MyoD during skeletal muscle development (Mueller et al., 2015). Based on the results of differential co-expression, the lncRNAs and mRNAs that are not on the same chromosome were selected as candidate targets. In the comparisons of A versus B, A versus C, A versus D, B versus C, B versus D, and C versus D, 29, 19, 24, 38, 8, and 47 pairs of LncRNAs and genes were found to have *trans*-regulation relations. These results suggest that lncRNA may participate in the development of Japanese flounder skeletal muscle through *cis*- or *trans*-acting mechanisms.

To identify the potential function of lncRNAs, in this study, we performed GO and KEGG enrichment analysis on the predicted *cis*- and *trans*-target genes of differentially expressed lncRNAs to understand the function of these differentially expressed lncRNA target genes and further predict the biological function of lncRNAs. In these enrichment analysis results, we find that the *cis*-acting regulatory gene LOC109635692 is enriched for the term “myosin VII complex, actin filament binding.” Furthermore, LOC109635692 is also found to be the *cis*-target gene predicted by lncRNA TCONS_00056200 and TCONS_00056180. The gene shows a downward trend in the comparison of A and B. During fish growth, increasing muscle fiber number or muscle size is the major muscle growth process. This gene is related to one of the factors that affect muscle development. A negative correlation is observed between LOC109635692 and TCONS_00056200, and a positive correlation is observed between LOC109635692 and TCONS_00056180, indicating that different lncRNAs may regulate the same target gene in different ways to regulate muscle development. Some coding genes regulated by lncRNA *trans*-action are also enriched in terms and pathways related to muscle development. For example, LOC109626851 identified in the comparison of B and C is enriched in the myosin filament term, LOC109648097 identified in the comparison of B and D is also enriched in the term of myosin filament, and the genes identified in the comparison of C and D, such as LOC109626999 and LOC109643555, are enriched in terms, such as actin binding. These genes are all *cis*- or *trans*-regulated with lncRNA. These results indicate that lncRNA may participate in the development of skeletal muscle in Japanese flounder.

Previous studies show that cacna1d is an important regulator of muscle development (Krasnyi and Ozernyuk, 2011, Park et al., 2018). In the lncRNA–gene network comparing A and C, the cacna1d gene -s the predicted *cis*-target of TCONS_00034769 (Figure 3B). In addition, it is enriched in the term of “skeletal muscle fiber development” in GO enrichment analysis. Moreover, the expression levels of cacna1d and TCONS_00034769 in stage A are higher than those in stage C. These results indicate that TCONS_00034769 targets cacna1d through a *cis*-regulatory mechanism to regulate skeletal muscle development in Japanese flounder. In the lncRNA–gene network comparing A and B, the actin-related protein 3B-like gene is the predicted *cis*-target of TCONS_00041871 (Figure 3A). In addition, it is enriched in the terms of “regulation of myosin II filament organization, positive regulation of actin filament polymerization, and actin binding” in GO enrichment analysis. In the skeletal muscle of Japanese flounder, the expression level of actin-related protein 3B-like in stage B is higher than that in stage A, indicating that this gene may be related to one of the factors that affect muscle development. The predicted regulatory lncRNA, TCONS_00041871, can control the expression of actin-related protein 3B-like through the *cis*-regulatory mechanism and is expressed at a higher level in skeletal muscle in stage A than in stage B. This negative correlation between LncRNA and target genes indicates that lncRNA regulates the muscle development of Japanese flounder by inhibiting the expression of the gene. Kin of IRRE like 2 (kirrel2) is predicted to be a *cis*-acting target of TCONS_00089031 (Figure 3A), and it is enriched in the terms of “myosin binding and positive regulation of actin filament polymerization” in GO enrichment analysis. In the skeletal muscle of Japanese flounder, the expression level of kirrel2 in stage A is higher than that in stage B. The predicted regulatory lncRNA, TCONS_00089031 can regulate the expression of kirrel2 through the *cis*-regulatory mechanism and is expressed at a higher level in skeletal muscle in stage A than in stage B. This positive correlation between lncRNA and target genes indicates that lncRNA regulates the muscle development of *Paralichthys olivaceus* by promoting the expression of the gene. In the lncRNA–gene network comparing A and D, the myosin-7B-like gene is the predicted *cis*-target of TCONS_00038291 (Figure 3C). In addition, it is enriched in the term of “myosin filament” in GO enrichment analysis. These results indicate that these lncRNAs play a role in the development of Japanese flounder skeletal muscle by regulating the related genes.

Both lncRNA and miRNA have their own regulatory networks. Their regulatory networks are not independent but are intertwined and interdependent, and they also have regulatory relationships and form complex regulatory networks with mRNA. In the study of pancreatic cancer, the large-scale effects of interrelated miRNAs are revealed by establishing an lncRNA–miRNA–mRNA regulatory network and constructing a model for predicting the disease mechanisms of miRNAs (Ye et al., 2014). In this study, we also predicted the biological function of lncRNAs by establishing lncRNA–miRNA–mRNA networks. Co-expression networks show that most lncRNAs interact with one or two predicted miRNAs that are involved in muscle growth and development. Some of these lncRNAs (including TCONS_00093971, TCONS_00096817, TCONS_00032744, etc.) have established interactions with at least three target miRNAs. Although these lncRNAs require further experimental validation, this information may help us explore the potential regulatory mechanisms of lncRNAs during Japanese flounder skeletal muscle growth and development.

## Data Availability Statement

The sequencing data obtained from the RNA-seq were released to the National Center for Biotechnology Information (NCBI) Sequence Read Archive (SRA) database under the accession numbers SRR12102079, SRR12102078, SRR12102077, SRR12102076, SRR12102075, SRR12102074, SRR12102073, SRR12102072, SRR12102071, SRR12102070, SRR12102069, and SRR12102068.

## Ethics Statement

The animal study was reviewed and approved by the Respective Animal Research and Ethics Committees of Ocean University of China.

## Author Contributions

SW finished the experiment, analyzed the data, designed the tables and figures, and drafted the manuscript. BL, JZ, YH, SL, MZ, JL, YL, and HW revised the manuscript. FH conceived the study and revised the manuscript. All authors contributed to manuscript revision and approved the submitted version.

## Conflict of Interest

The authors declare that the research was conducted in the absence of any commercial or financial relationships that could be construed as a potential conflict of interest.

The reviewer QZ declared a shared affiliation, with no collaboration, with several of the authors, FH, SW, JZ, BL, YH, SL, HW, MZ, JL, YL, to the handling editor at the time of review.

## Footnotes

1 https://www.ncbi.nlm.nih.gov/genome/?term=Japanese+flounder

2 http://bioconductor.org/packages/release/bioc/html/DESeq.html
