# Supplementary material for: Identification and Characterization of lncRNAs Related to the Muscle Growth and Development of Japanese Flounder (Paralichthys olivaceus)
Source: Front Genet. 2020 Sep 9;11:1034. doi: 10.3389/fgene.2020.01034 (PMC7510837; doi:10.3389/fgene.2020.01034)
Supplement: Supplementary file 1 [file Data_Sheet_1.zip › Suppl. files/Figure S1.docx]

**Figure S1A**

**
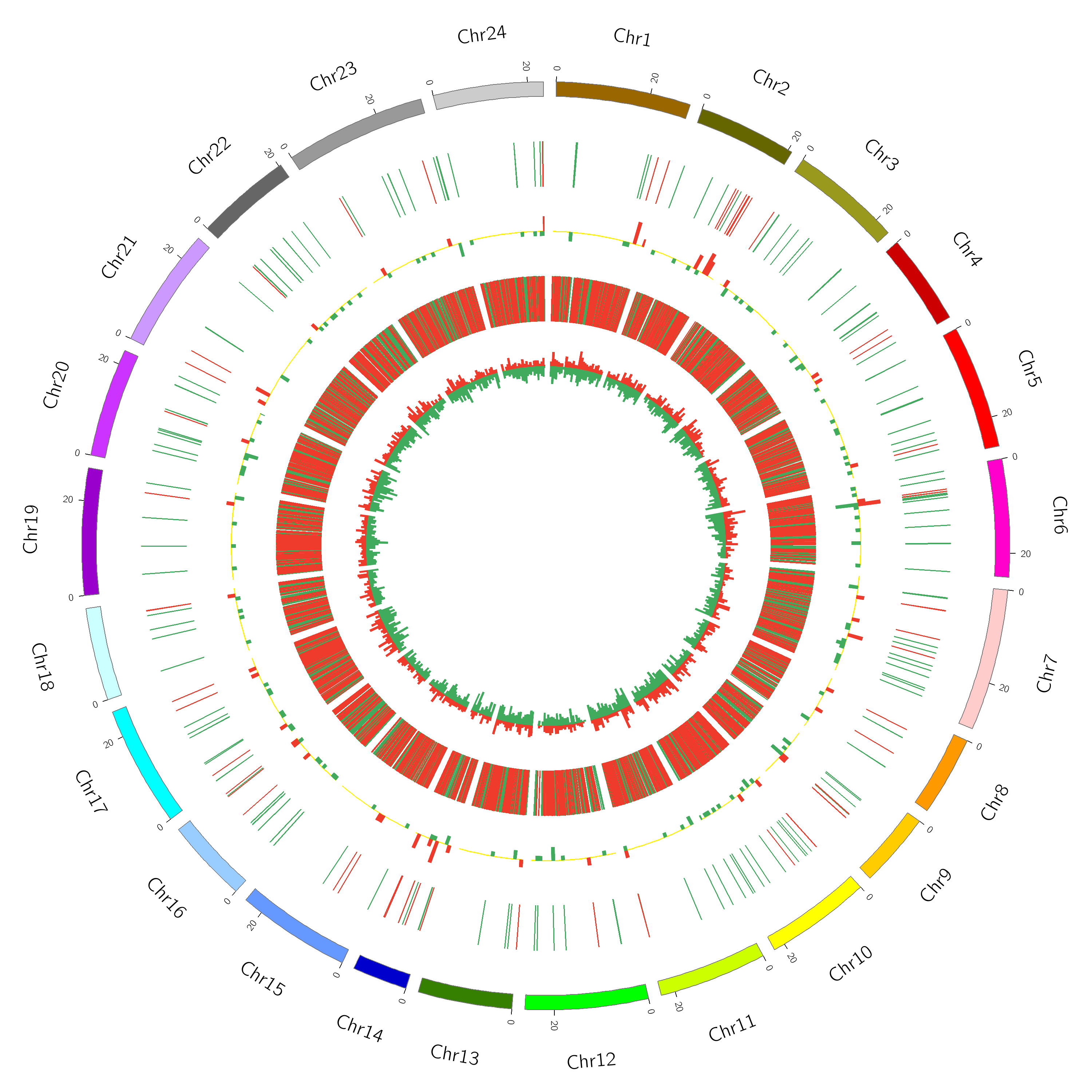
**

**Figure S1B**

**
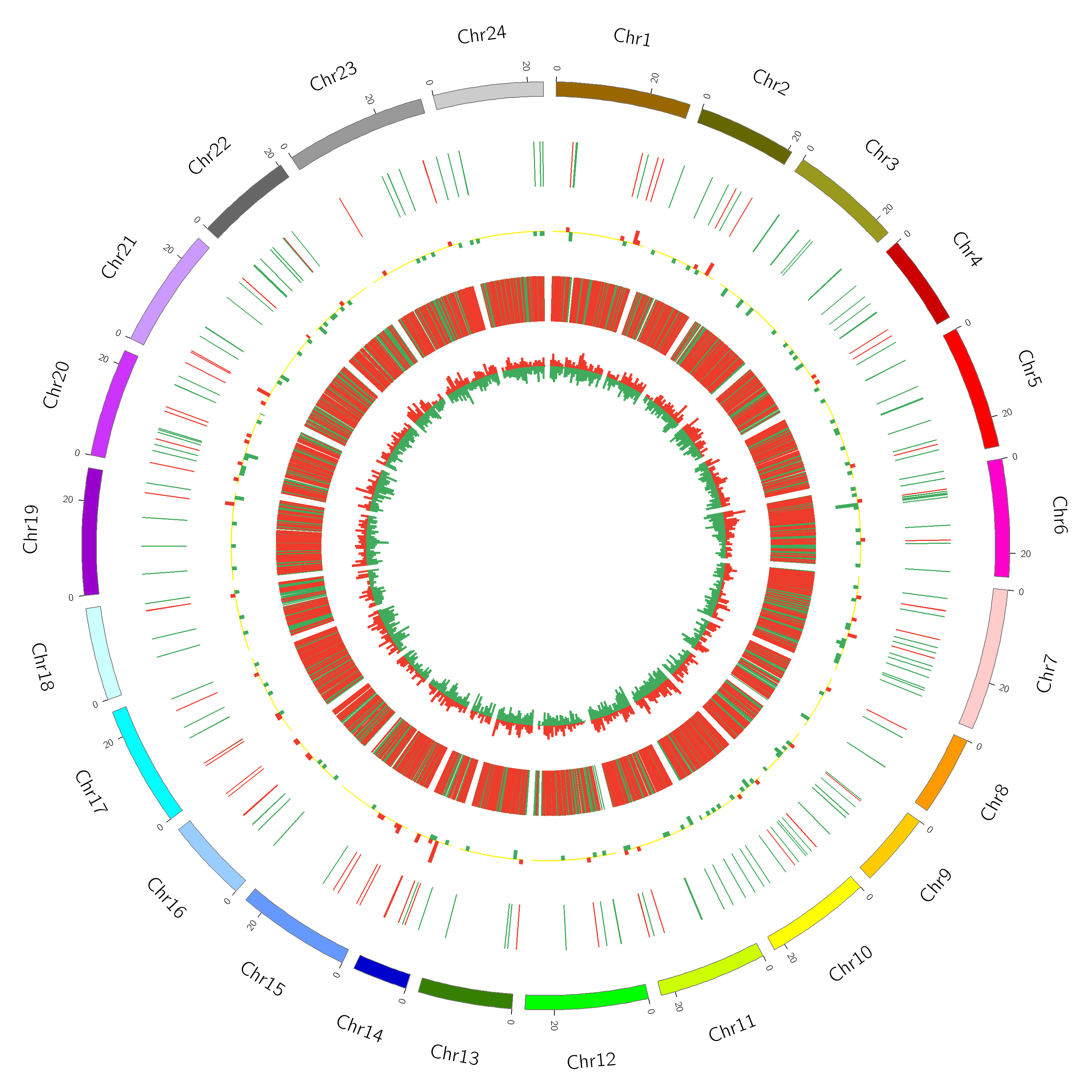
**

**Figure S1C**

**
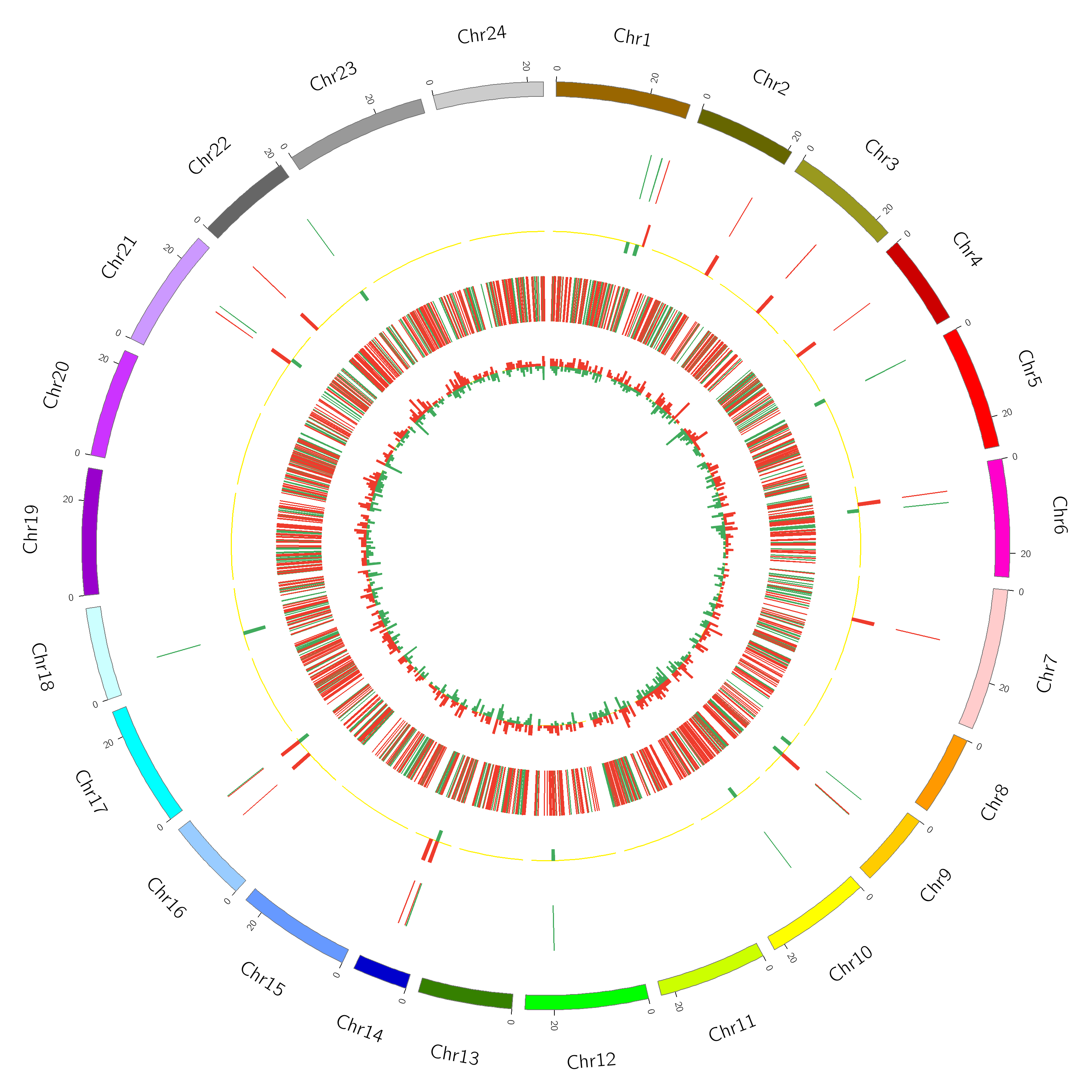
**

**Figure S1D**

**
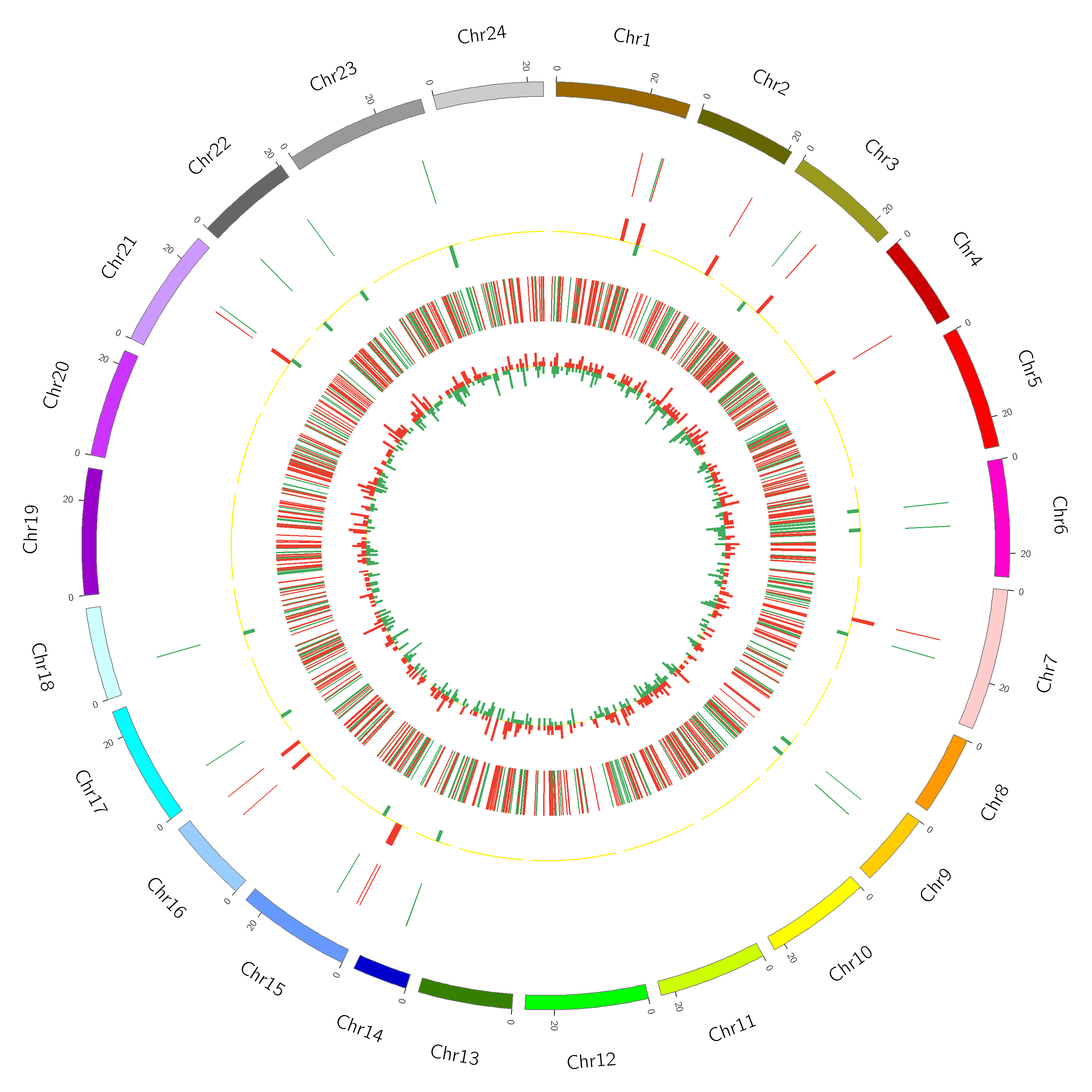
**

**Figure S1E**

**
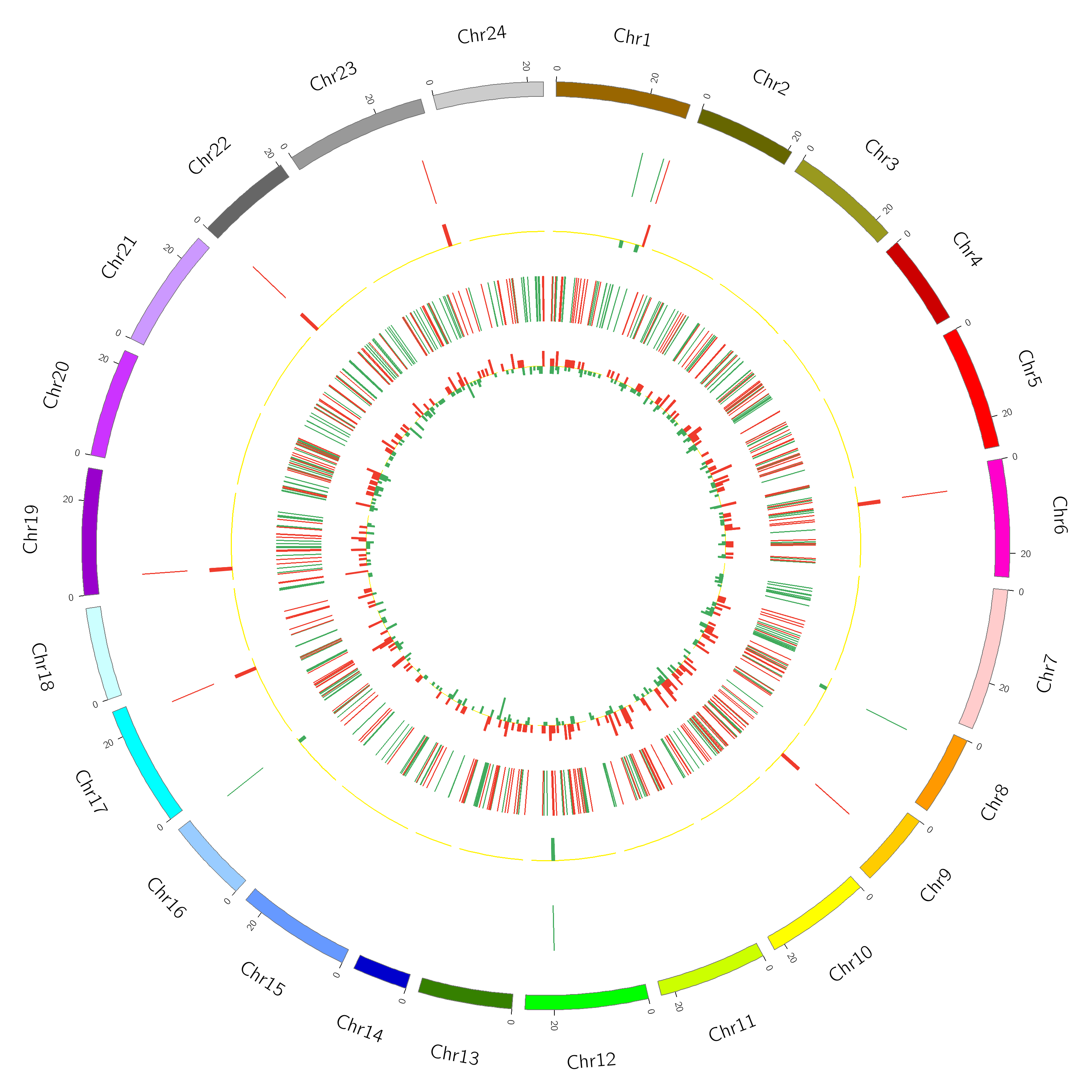
**

Figure S1

Circos diagram of differential expression of lncRNA and mRNA. (S1A) A vs. C; (S1B) A vs. D; (S1C) B vs. C; (S1D) B vs. D; (S1E) C vs. D. In the figure, the outermost circle is the autosomal distribution of the Japanese flounder; the second circle is the lncRNA of differential expression on the chromosome, the red line indicates up-regulation, the green line indicates down-regulation; the third circle is the histogram of differentially expressed lncRNAs at different positions. Red indicates up-regulation, green indicates down-regulation, and the higher the column, indicates the more differentially expressed gene numbers. The fourth circle is the distribution of differentially expressed mRNAs on the chromosome, and the color distribution is the same as lncRNA; the innermost circle is the column with differentially expressed mRNAs at different positions, color distribution is the same as lncRNA.
