# Supplementary material for: Identification and Characterization of lncRNAs Related to the Muscle Growth and Development of Japanese Flounder (Paralichthys olivaceus)
Source: Front Genet. 2020 Sep 9;11:1034. doi: 10.3389/fgene.2020.01034 (PMC7510837; doi:10.3389/fgene.2020.01034)
Supplement: Supplementary file 1 [file Data_Sheet_1.zip › Suppl. files/Figure S2.docx]

**Figure S2A**

**
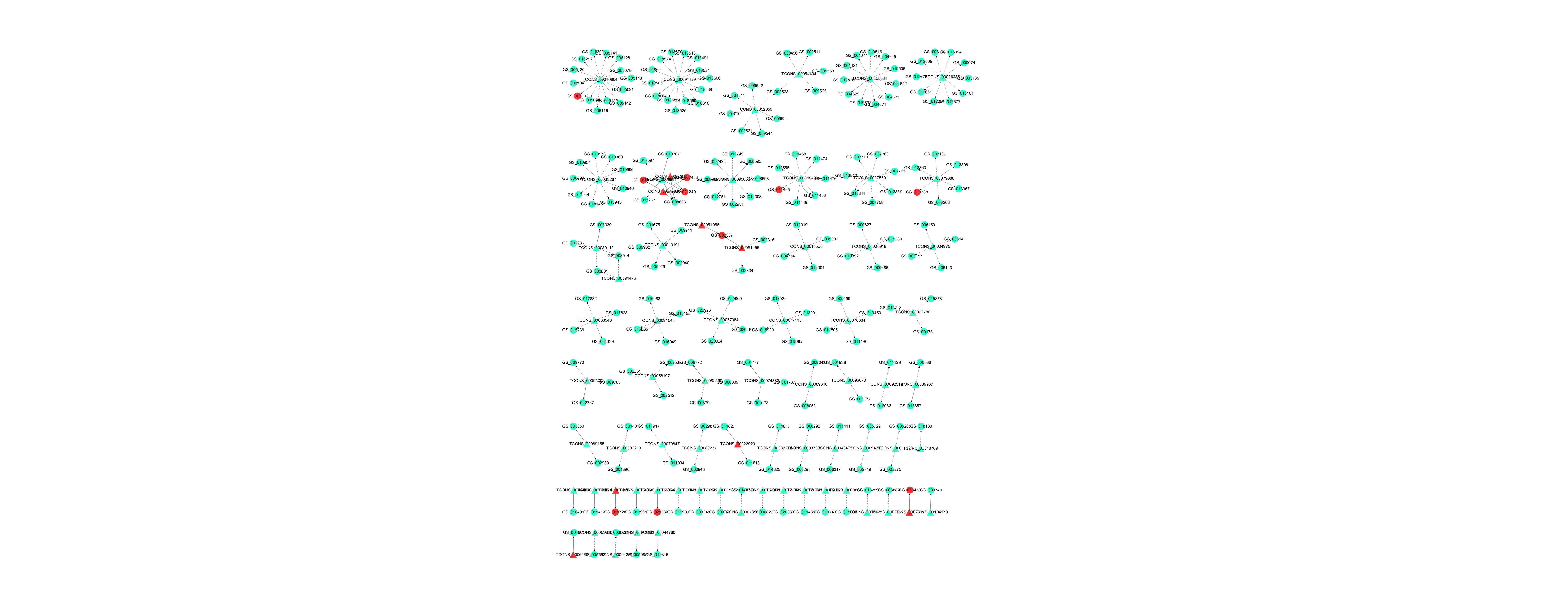
**

**Figure S2B**

**
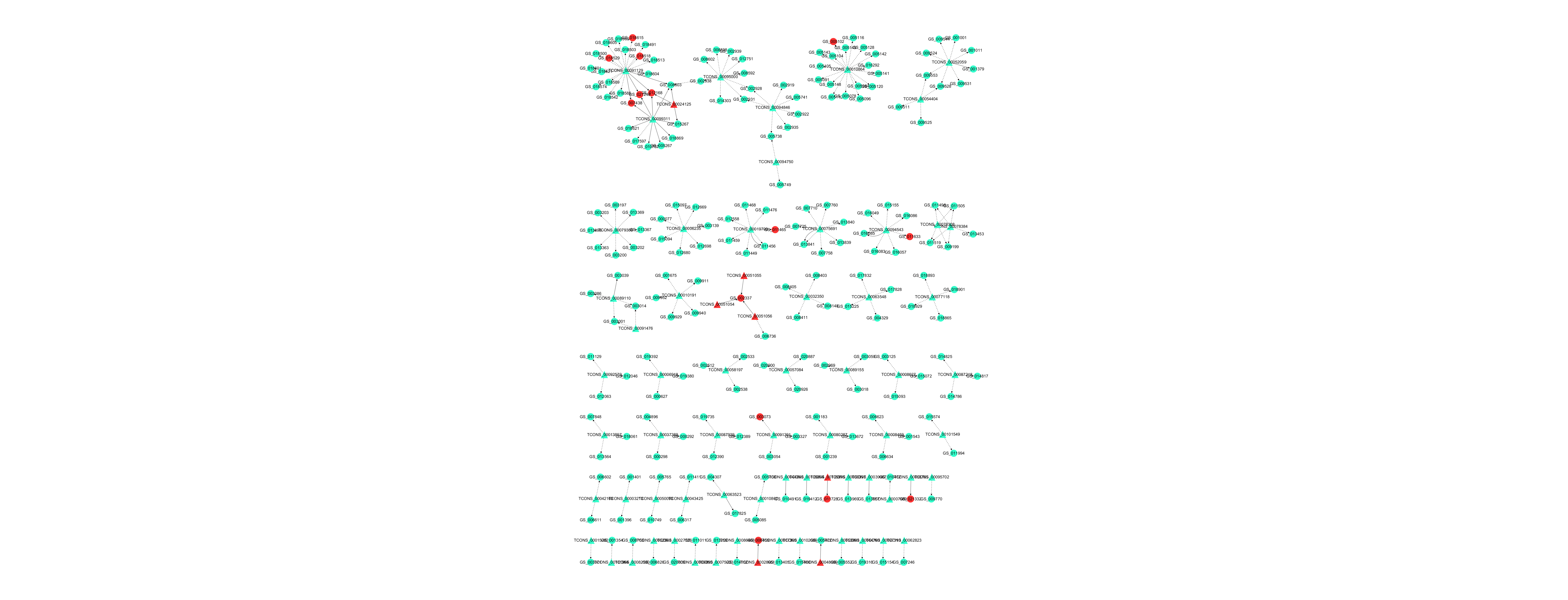
**

**Figure S2C**

**
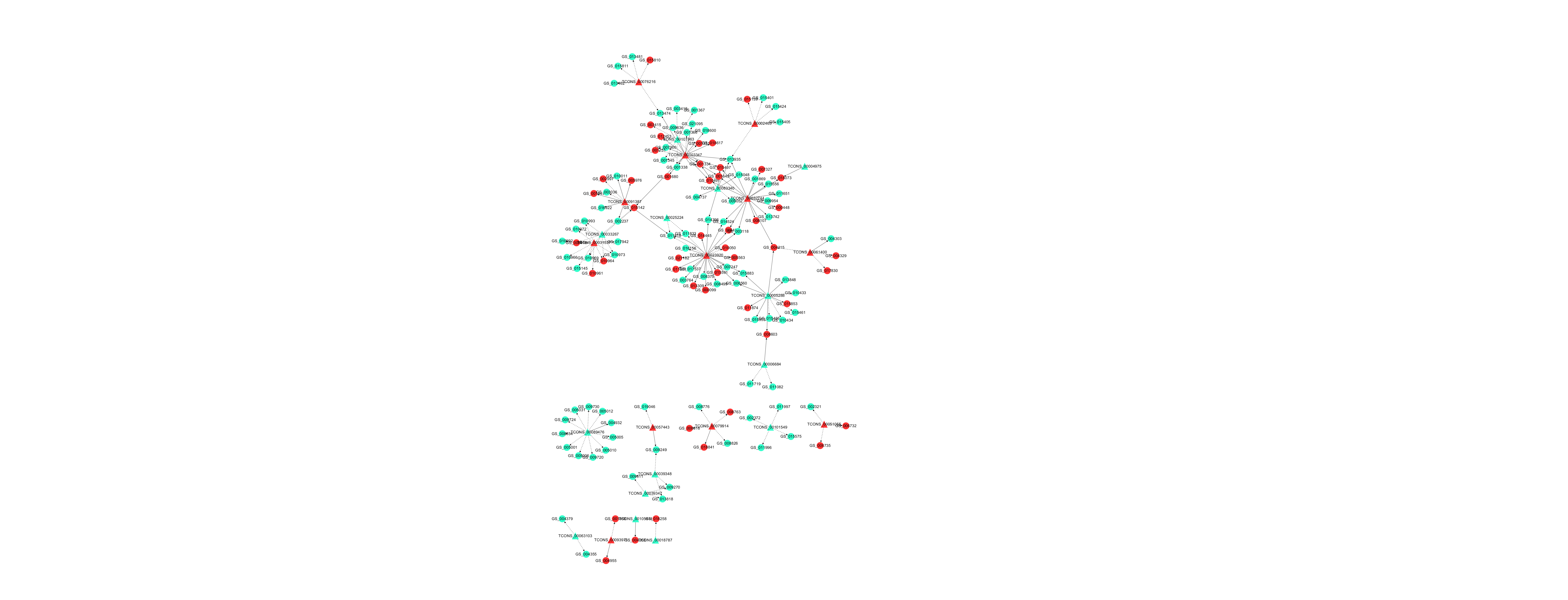
**

**Figure S2D**

**
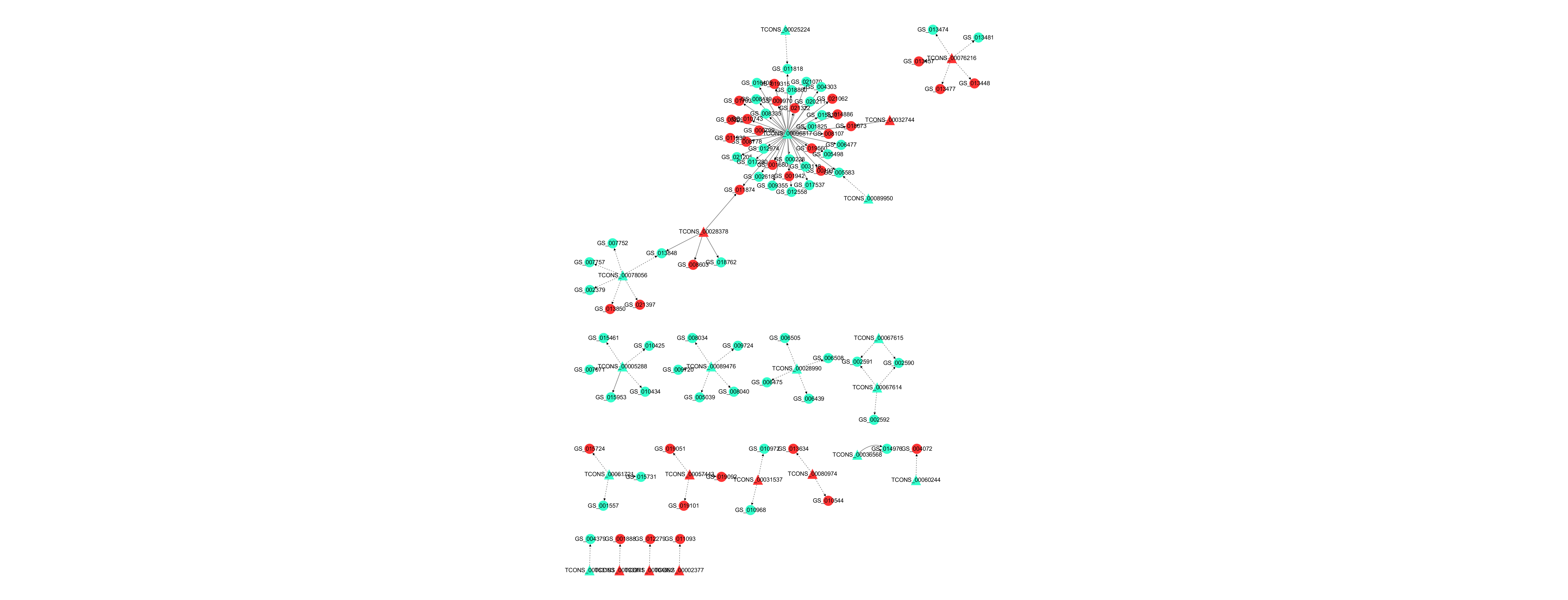
**

**Figure S2E**

**
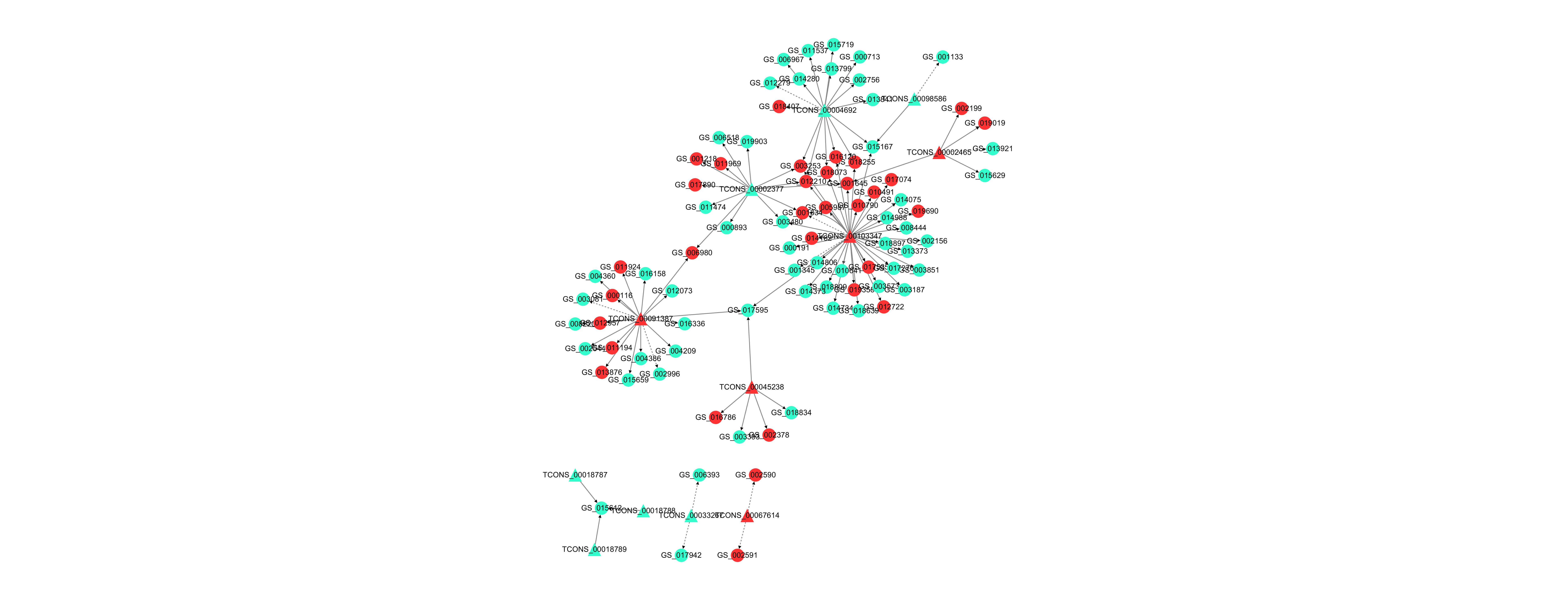
**

Figure S2

LncRNA - gene interaction network diagram. (S2A) A vs. C; (S2B) A vs. D; (S2C) B vs. C; (S2D) B vs. D; (S2E) C vs. D. Red indicates up-regulation, green indicates down-regulation, triangles represent lncRNA, and circles indicate mRNA. The dashed line indicates the interaction between the differentially expressed lncRNA and its corresponding cis target gene, while the solid line indicates the interaction between the differentially expressed lncRNA and its corresponding trans target gene.
