# Supplementary material for: Identification and Characterization of lncRNAs Related to the Muscle Growth and Development of Japanese Flounder (Paralichthys olivaceus)
Source: Front Genet. 2020 Sep 9;11:1034. doi: 10.3389/fgene.2020.01034 (PMC7510837; doi:10.3389/fgene.2020.01034)
Supplement: Supplementary file 1 [file Data_Sheet_1.zip › Suppl. files/Figure S3.docx]

**Figure S3A**


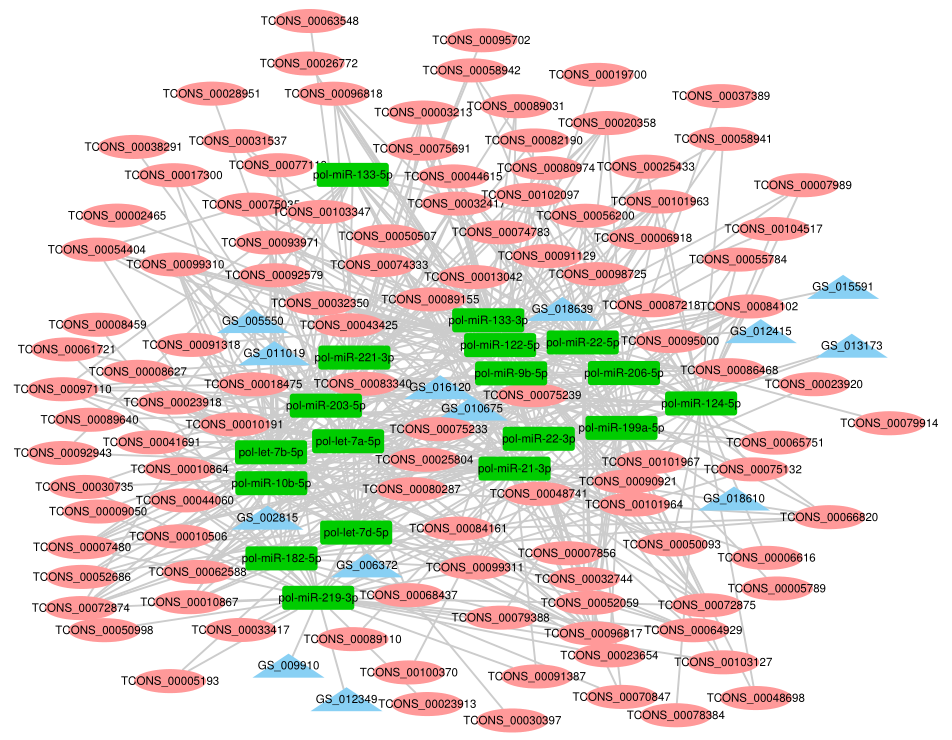


**Figure S3B**


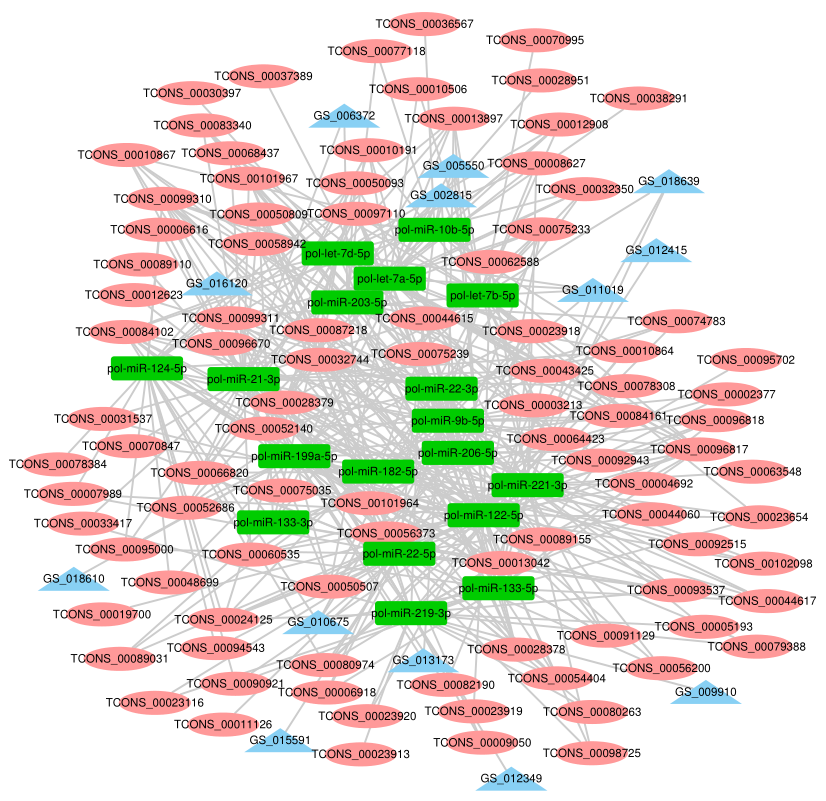


**Figure S3C**


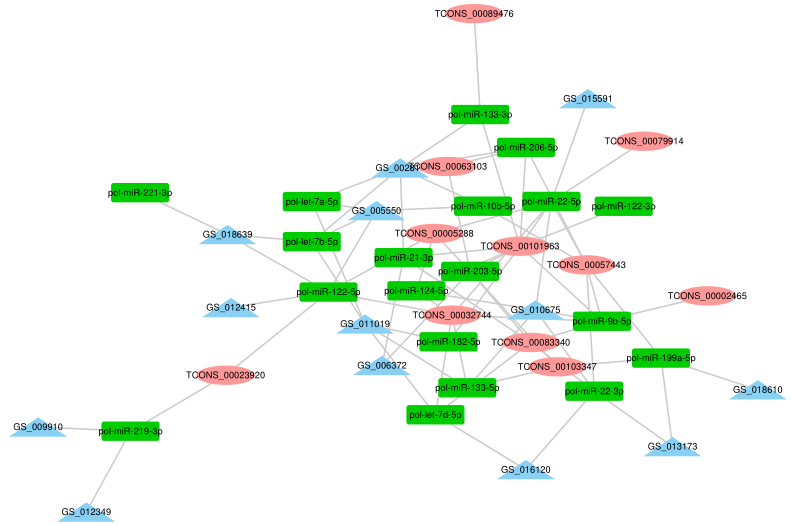


**Figure S3D**


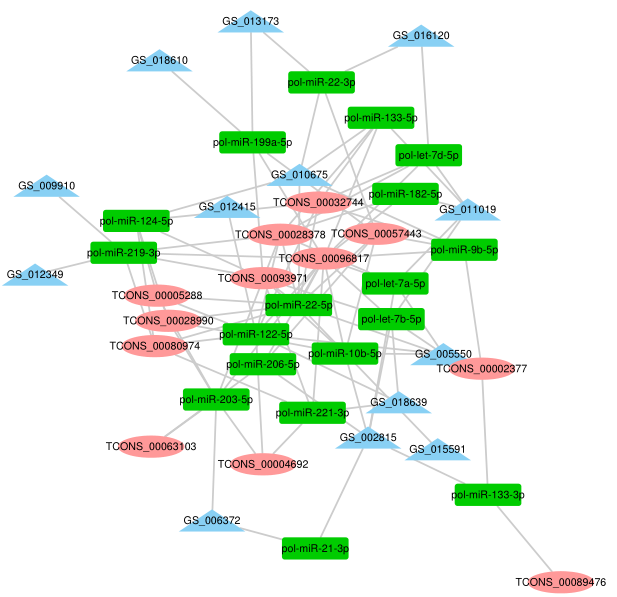


**Figure S3E**


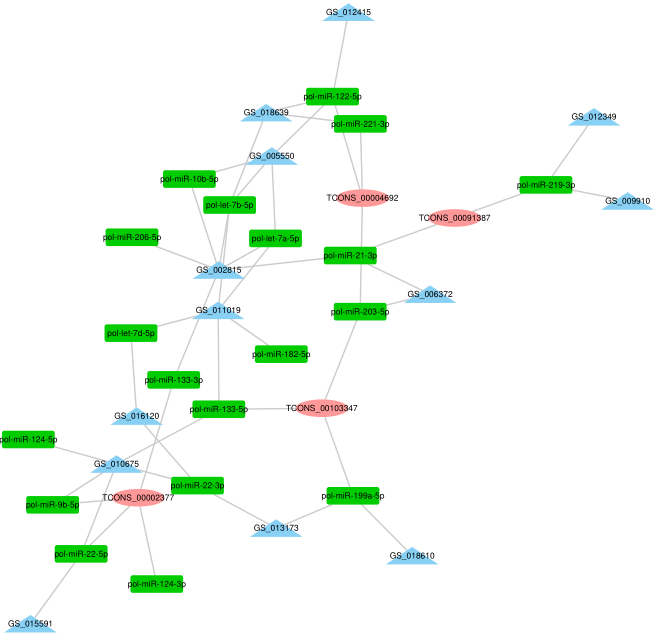


Figure S3

The lncRNA - miRNA-mRNA interaction network. (S3A) A vs. C; (S3B) A vs. D; (S3C) B vs. C; (S3D) B vs. D; (S3E) C vs. D. In the network, blue triangles represent mRNAs, red ovals represent lncRNAs, and green rectangles represent miRNAs.
